# Magnoflorine Ameliorates Inflammation and Fibrosis in Rats With Diabetic Nephropathy by Mediating the Stability of Lysine-Specific Demethylase 3A

**DOI:** 10.3389/fphys.2020.580406

**Published:** 2020-12-22

**Authors:** Liang Chang, Qi Wang, Jiannan Ju, Yue Li, Qiao Cai, Lirong Hao, Yang Zhou

**Affiliations:** Department of Nephrology, The First Affiliated Hospital of Harbin Medical University, Harbin, China

**Keywords:** diabetic nephropathy, magnoflorine, renal fibrosis, lysine-specific demethylase 3A, transforming growth factor β-induced factor-1

## Abstract

Diabetic nephropathy (DN) represents one of the most devastating complications for patients with diabetes. The anti-diabetic activities of Magnoflorine (MF) were reported, with underlying mechanism unknown. Lysine-specific demethylase 3A (KDM3A) was identified in the renal injuries. In the current study, we investigated the functional role of MF in DN progression with the involvement of KDM3A. We reported that in the animal model of DN induced by streptozotocin (STZ) injection, MF attenuated inflammatory response and fibrosis in the kidneys. In cultured mesangial cells, MF similarly ameliorated abnormal proliferation and lowered the expression of inflammation- and fibrosis-related factors stimulated by high glucose (HG) treatment. Upon MF treatment, there was a decline in KDM3A-positive cells in renal tissues of rats, accompanying an augment in KDM3A ubiquitination. KDM3A upregulation *in vitro* by a proteasome inhibitor MG132 comparably dampened the inhibitory role of MF in inflammatory response and fibrosis. Further analyses revealed that MF increased transforming growth factor β-induced factor 1 (TGIF1) transcriptional activity by promoting ubiquitination and degradation of KDM3A, thus inhibiting the activation of TGF-β1/Smad2/3 signaling pathway. TGIF1 silencing weakened the repressive role of MF in mesangial cells as well. In conclusion, MF contributes to TGIF1 transcription *via* an epigenetic mechanism.

## Introduction

The incidence and pervasiveness of diabetes mellitus have grown remarkably throughout the world, and the global increase in the amount of people with diabetes contributed a lot to the surge of diabetic kidney disease (Tuttle et al., 2014). The etiology of diabetic kidney disease is complicated and involved many factors, including genetic factors, hypertension, abnormalities of renal hemodynamics, as well as metabolism of vasoactive substances (Lu et al., 2019). Diabetic nephropathy (DN), the major cause of chronic kidney disease, is characterized by albuminuria, lowered glomerular filtration rate, hypertension, and mesangial matrix expansion in addition to tubulointerstitial fibrosis (Giralt-Lopez et al., 2020). The progression of DN has been suggested to attribute to various pathological mechanisms, including inflammation and fibrosis (Calle and Hotter, 2020). The current therapeutic maneuver of diabetic patients focuses on glycemic control and antihypertensive/lipidlowering treatments; however, these involvements do not prevent the development of chronic kidney disease in most diabetic patients (Rayego-Mateos et al., 2020). Therefore, the development for novel therapeutic approaches against DN is an area of great importance.

Magnoflorine (MF), an aporphine alkaloid exists in plants belonging to the Berberidaceae, Magnoliaceae, Menispermaceae, or Papaveraceae botanical families, has been indicated to exert antioxidant, anti-inflammatory, and anti-diabetic functions (Okon et al., 2020). Moreover, MF reduced the expression patterns of pro-inflammatory cytokines tumor necrosis factor alpha (TNF-α) and interleukin (IL)-1β, thus ameliorating acute lung injury induced by lipopolysaccharide (Guo et al., 2018). On the basis of the existing reports, we believed that MF exerts a protective role against DN possibly through its anti-inflammatory properties. However, whether it also exerts an anti-fibrotic role, and if so, how that was achieved remains an enigma. Honokiol, a natural phenolic compound separated from Magnolia officinalis, could induce the degradation of an oncoprotein AML1-ETO concentration- and time-dependently in leukemic cells (Zhou et al., 2017). Since MF and Honokiol both have the basic structure of o-methyl-linked phenol, it is assumed that MF and Honokiol have similar functions. Therefore, we believed that MF is participated in the DN development through a similar manner. Interestingly, lysine-specific demethylase 3A (KDM3A) enhanced transforming growth factor *β*-induced factor 1 (TGIF1), inhibited fibronectin, alpha skeletal muscle actin (*α*-SMA) as well as the phosphorylation of Smad2/3 to curtail renal fibrosis (Ding et al., 2020). In addition, ectopic expression of TGIF1 remarkably inhibited Smad-mediated activation of TGF-*β*1 and blocked α-SMA expression induced by TGF-β1 (Dai and Liu, 2004). As a consequence, we postulated that MF mediated the degradation of KDM3A to regulate the expression of TGIF1. Pathological features of DN, such as glomerular basement membrane thickening, glomerular membrane dilatation, glomerulosclerosis, and tubulointerstitial fibrosis are closely associated with disease progression (Wolf, 2004). The main components of the glomerulus are the mesangial cells and stroma that support its structure, function, and regulation (Wolf, 2004). Hyperglycemia stimulates the production of cytokines and growth factors by glomerular mesangial cells and promotes the expression of collagen IV and fibronectin, which then gradually alters glomerular structure until DN occurs (Schena and Gesualdo, 2005). In addition, the high glucose (HG)-treated mesangial cell line MES13 cells were used to model DN (Kim et al., 2019). The MES13 cell line was also used to study the role of dibenzoylmethane in the treatment of DN (Lee et al., 2019). Hence, in this study, we created a DN rat model using streptozotocin (STZ) and a cell model using the SV40-MES13 murine mesangial cell line to evaluate the therapeutic effects of MF and to decipher possible anti-inflammatory and anti-fibrotic mechanisms. This study might provide a theoretical basis for the application of MF in controlling DN.

## Materials and Methods

### Animals

All animal experiments were reviewed and approved by the Ethics Committee of the First Affiliated Hospital of Harbin Medical University. Twenty male Sprague Dawley rats (6–8 weeks old) were purchased from SLAC Co., Ltd. (Shanghai, China) and maintained in a specific-pathogen-free environment. To induce DN, rats were injected intraperitoneally with STZ (Sigma-Aldrich Chemical Company, St Louis, MO, United States) in 0.1 M citrate buffer at 70 mg/kg. The controls rats (sham group) were only injected with citrate buffer. Blood glucose levels were then checked until the blood glucose levels reached 16.7 mmol/L (defined as diabetes). Subsequently, we measured proteinuria levels in rats daily for 4–6 weeks after STZ induction, and we defined 24-h proteinuria greater than 30 mg/24 h as DN in rats (Bai et al., 2019). Subsequently, diabetic rats were orally administered with MF dissolved in phosphate buffered saline (PBS; DN + MF group) or PBS alone (DN + PBS group) at 100 mg/kg for 30 days.

Blood samples were centrifuged at room temperature. Levels of blood urea nitrogen (BUN), Cys-C and serum creatinine (Scr) in the serum were analyzed using a Roche Modular P800 analyzer (Roche Diagnostics, Indianapolis, IN, United States) by alkaline picric acid. After blood sample collection, rats were euthanized by an intraperitoneal injection of pentobarbital sodium. The death of rats was confirmed by no heartbeat (excluding cardiac arrest), no nerve reflex, and no blink reflex. Subsequently, a portion of the collected left renal tissues was fixed with 10% formalin for histological staining. Right renal tissues were harvested for messenger RNA (mRNA) and protein detection.

### Histological Staining

The renal tissues were fixed in 4% paraformaldehyde for 24 h and then embedded in paraffin. Renal tissues were cut into 5-μm-thick slices to visualize the cell structure. Hematoxylin-eosin (HE) staining and Masson’s trichrome staining were then performed to assess basic tissue structure and detect fibrosis. Photos were taken under a Leica microscope (DM2500, Wetzlar, Germany). The distribution of collagen was measured by two staining methods (Masson’s trichrome staining and picrosirius red staining). Histopathological sections were analyzed using Image-Pro Plus software (version 6.0, National Institutes of Health, Bethesda, MD, United States). Collagen area was calculated as positive collagen staining area divided by whole renal tissue (%).

### Enzyme-Linked Immunosorbent Assay

Murine enzyme-linked immunosorbent assay (ELISA) kits for TNF-α and IL-1β were from BOSTER Biological Technology Co., Ltd. (Wuhan, Hubei, China) for the assessment of their protein expression in mesangial cells. Mesangial cells (6 × 10^5^) were grown in a 6-well plate for 48 h to collect the medium supernatant. The diluted samples and standards at 1,000, 500, 250, 125, 62.5, 31.3, or 15.6 μg/ml were added to the ELISA plate. The sample was sealed with a sealed film for 1.5 h, incubated with antibodies against IL-1β or TNF-*α* for 1 h, and reacted with 100 μl prepared ABC working solution for 30 min (all at 37°C). Subsequently, the samples were subjected to a staining with 3,3',5,5'-tetramethylbenzidine substrates until a clear blue gradient appeared. Optical density (OD) values were assessed at 450 nm by a microplate reader to plot the standard curve, on which the protein expression was determined.

### Immunohistochemistry

Renal tissues were fixed with 10% formalin and embedded in paraffin, and the embedded samples were cut into 5 μm sections and fixed on slides. After antigen retrieval, sections were incubated with antibodies against fibronectin, α-SMA, KI67, or CD163 overnight at 4°C and with horseradish peroxidase-labeled secondary antibody [1:200; Cell Signaling Technologies (CST), Beverly, MA, United States]. Fibronectin and α-SMA expression could be observed using diaminobenzidine staining.

### RNA Isolation and Quantification

Total RNA was isolated from different groups of mesangial cells and tissues using TRIzol reagents (Invitrogen) on the basis of the manufacturer’s instructions. Then, the SuperScript™ IV one-step RT-PCR system (catalog number: 12594025, Invitrogen Inc., Carlsbad, CA, United States) was used to prepare the cDNA. Reverse transcription-quantitative polymerase chain reaction (RT-qPCR) reaction was conducted using QuantStudio®3 quantitative real-time PCR instruments (Applied Biosystems, Inc., Foster City, CA, United States). The amplification was performed using TaqMan™ Fast Advanced Master Mix (catalog number: 4444556, Applied Biosystems) with a total volume of 25 μl. The expression of indicated genes was analyzed through 2^-*Δ*ΔCt^. ABI Prism 7000SDS software (Applied Biosystems) was utilized to analyze data. The primer sequences are listed in Table 1.

**Table 1:** Primers list.

Gene	Forward (5'-3')	Reverse (5'-3')
IL-1β	TGGACCTTCCAGGATGAGGACA	GTTCATCTCGGAGCCTGTAGTG
TNF-α	GGTGCCTATGTCTCAGCCTCTT	GCCATAGAACTGATGAGAGGGAG
Fibronectin	ACAACACCGAGGTGACTGAGAC	GGACACAACGATGCTTCCTGAG
α-SMA	TGCTGACAGAGGCACCACTGAA	CAGTTGTACGTCCAGAGGCATAG
Collagen IV	ATGGCTTGCCTGGAGAGATAGG	TGGTTGCCCTTTGAGTCCTGGA
TGF-β1	TGATACGCCTGAGTGGCTGTCT	CACAAGAGCAGTGAGCGCTGAA
KDM3A	GCCAGCCTTAAAGGAAGACCTG	ACACAGCCACTGGCTCCAAAAC
TGIF1	CAGATTCTGCGAGACTGGCTGT	CGGGCGTTGATGAACCAGTTAC
GAPDH	GTCTCCTCTGACTTCAACAGCG	ACCACCCTGTTGCTGTAGCCAA

TGIF1, transforming growth factor β-induced factor 1; KDM3A, lysine-specific demethylase 3A; TNF, tumor necrosis factor; IL, interleukin; α-SMA, alpha skeletal muscle actin.

### Western Blot

Protein expression was detected after extraction of proteins in tissues and cells using radio immunoprecipitation assay buffer (Invitrogen). After being separated by sodium dodecyl sulfate polyacrylamide gel electrophoresis, the protein was transferred to a polyvinylidene fluoride membrane (Millipore Corp, Billerica, MA, United States). After being sealed with 5% skim milk for 2 h, the membrane was incubated with primary antibodies at 4°C overnight, followed by 2-h incubation with corresponding rabbit secondary antibody (1:4,000, Abcam, Cambridge, United States) at 37°C. Immunoreactive proteins were visualized using enhanced chemiluminescence (Millipore). The primary antibodies included α-SMA (1:1,000; Abcam), fibronectin (1:1,000; Abcam), collagen IV (1:1,000; Abcam), p53-upregulated modulator of apoptosis (PUMA, 1:1,000, #24633, CST), apoptotic protease activating factor 1 (Apaf1, 1:1,000, ab2001, Abcam), B-cell CLL/lymphoma 2 (Bcl-2, #3498, CST), TGF-β1 (1:1,000, #MA5-15065, Thermo Fisher), and glyceraldehyde-3-phosphate dehydrogenase (GAPDH, 1:1,000; Abcam).

### Cell Culture and Treatment

Mesangial cell line (SV40-MES13) from American Type Culture Collection (Manassas, VA, United States) were grown in Dulbecco’s modified Eagle’s medium containing 20% fetal bovine serum under 5% CO_2_ at 37°C. Cells were then stimulated with 5.5 mmol/L d-glucose plus 19.5 mmol/L mannitol (low glucose group; LG group), or 25 mmol/L glucose (HF group; Qin et al., 2017). Subsequently, we treated mesangial cells under HG condition with 100 μg/ml MF. After 48 h of treatment, the collected cells were used for following experiments.

### Collagen Content Detection

The content of collagen in rat kidney tissues was detected using a Total Collagen Assay kit (ab222942, Abcam) according to the instructions.

### Chromatin Immunoprecipitation

Chromatin immunoprecipitation (ChIP) assays were performed using the Pierce’s Magnetic ChIP Kit (Thermo Fisher Scientific) according to the manufacturer’s instructions. Briefly, mesangial cells treated or untreated with MF were used. Cells were cross-linked with 1% formaldehyde for 10 min at room temperature, followed by nucleus separation in lysis buffer and sonication. After centrifugation, the supernatant, ChIP grade H3K9me2 antibody (Abcam), and protein G magnetic beads were used for immunoprecipitation of chromatin. Normal rabbit antibody against IgG was used as a negative control. Finally, the purified input and immunoprecipitated DNA were analyzed by PCR using a PCR kit (Promega).

### Cell Counting Kit-8

Cell counting kit-8 (CCK-8) kits (Beyotime Institute of Biotechnology, Shanghai, China) were used to determine the viability of mesangial cells. Briefly, mesangial cells (3 × 10^3^ cells/well) were seeded into 96-well plates and cultured. A total of 20 μl CCK-8 was added at the specified time points. The OD value was detected at 450 nm by a microplate reader (550, Bio-Rad Laboratories, Hercules, CA, United States) after a 2-h incubation at 37°C.

### 5-Ethynyl-2'-Deoxyuridine Staining

Ethynyl-2'-deoxyuridine (EdU) cell proliferation detection kits (Ruibo Biotechnology Co., Ltd., Guangzhou, Guangdong, China) were applied for EdU analysis (cell proliferation). Briefly, cells were labeled with EdU, fixed with 4% paraformaldehyde, and then stained with 4',6-diamidino-2-phenylindole (DAPI). EdU fluorescence (red) was detected by a Nikon A1R laser scanning confocal microscope (NIKON, Tokyo, Japan).

### Apoptosis Detection

The cells were placed in cold 70% ethanol and stained at room temperature in dark with Annexin V-fluorescein isothiocyanate (FITC)/propidium iodide (PI) for 15 min. Apoptosis was detected immediately using FACScan (Beckman Coulter, Fullerton, CA, United States) and analyzed using CellQuest software.

### LDH Release Detection

In brief, the mesangial cells were treated as described above and centrifuged at 2,000 *g* for 20 min. The lactate dehydrogenase (LDH) release was evaluated using an LDH assay kit according to the protocol. Thereafter, the OD value was detected at wavelength of 490 nm, and all values of % LDH released were normalized to the untreated control group.

### Immunofluorescence Staining

The mesangial cells immobilized in 4% paraformaldehyde were mixed with 0.1% Triton X-100 for 15 min, blocked in 5% bovine serum albumin (BSA) for 1 h and treated with the primary antibodies against fibronectin and α-SMA for 1 h at 37°C. After DAPI staining, a laser confocal microscopy was used for observation.

### Co-immunoprecipitation

Mesangial cell (2 × 10^7^ cells) were dissolved in lysis buffer and incubated overnight with 2 μg antibody against Ubiquitin or KDM3A. To reduce nonspecific binding and background, 40 μl protein A coupled agarose beads (Sigma-Aldrich) were incubated with 0.1% BSA for 1 h. The pre-cleared protein A beads were added to the mixture (total protein + antibody) for a 4-h rotation at 4°C. After incubation, the beads were washed with lysis buffer and eluted with Laemmli buffer.

### Statistical Analysis

All data from three independent assays are displayed in the form of means ± SD. Unpaired *t*-test, one-way or two-way ANOVA, followed by Tukey’s *post hoc* test was utilized for comparison between two groups or multiple groups, respectively. Statistical significance was reflected at *p* < 0.05.

## Results

### MF Attenuates STZ-Induced Inflammatory Response and Abnormal Hyperplasia in Rats

**Figure 1:**
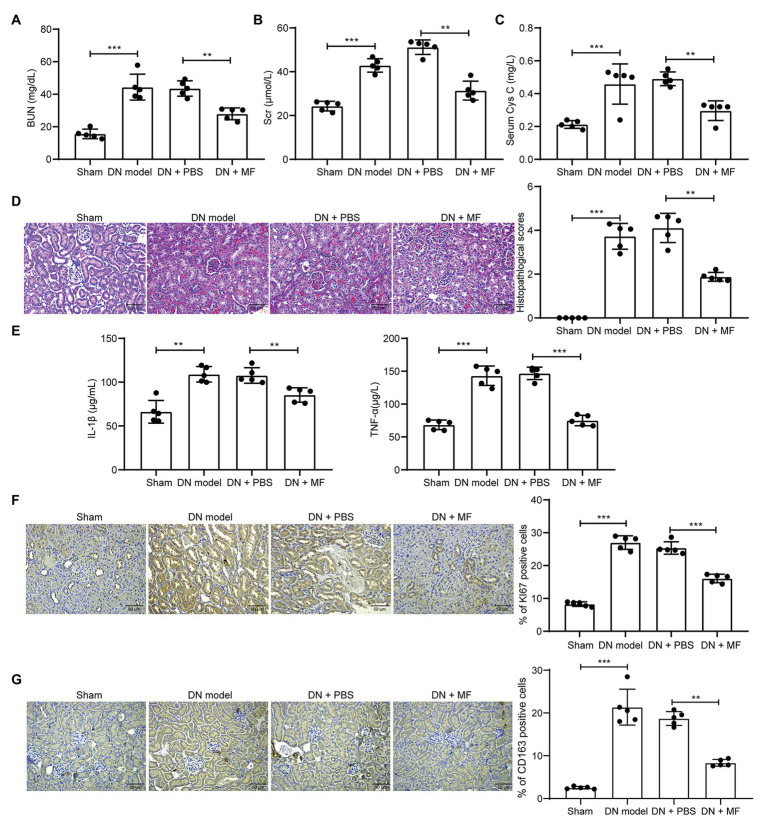
Magnoflorine (MF) attenuates streptozotocin (STZ)-induced abnormal hyperplasia and inflammatory response in rats. **(A)** serum levels of blood urea nitrogen (BUN) in renal tissues of rats; **(B)** serum levels of serum creatinine (Scr) in renal tissues of rats; **(C)** serum levels of Cys-C in renal tissues of rats; **(D)** hematoxylin-eosin (HE) staining of pathological structure of rat renal tissue sections; **(E)** enzyme-linked immunosorbent assay (ELISA) detection of interleukin (IL)-1β and tumor necrosis factor alpha (TNF-α) levels in rat serum; **(F)** immunohistochemical staining of KI67 positive cells in renal tissues; **(G)** immunohistochemical staining of CD163 positive cells in renal tissues. All experiments were repeated three times. Values are expressed as mean ± SD (*n* = 5, each dot represents a rat in **A–C** and **E–G**). Variance among more than two groups was analyzed by one-way (**A**,**B**,**C**,**F**,**G**) or two-way ANOVA (**D**), followed by Tukey’s *post hoc* test. ^**^p < 0.01, ^***^p < 0.001.

We first collected serum from rats and detected levels of kidney injury-related factors, such as BUN, Scr, and Cys-C in the serum. We found that 100 mg/kg MF significantly decreased the expression of these factors (Figures 1A–C) in rat serum. Subsequently, we euthanized the rats and extracted the renal tissues. We found that STZ-treated rats had obvious hyperplasia, increased infiltration of inflammatory cells, and blurred space between the cells (Figure 1D). We further observed that the levels of IL-1β and TNF-α in rat serum were reduced by MF (Figure 1E) in rats. However, after the treatment of rats with MF, the tissue hyperplasia in the kidney and the infiltration of inflammatory cells were obviously reduced, and the pathological structure of the renal tissues was improved. We also detected the expression of KI67 and macrophage marker protein CD163 in tissues by immunohistochemistry and found that the number of KI67 and CD163 positive cells was increased significantly after STZ treatment, while inhibited after further treatment with MF (Figures 1F,G).

### MF Attenuates STZ-Induced Fibrosis in Rat Renal Tissues

**Figure 2:**
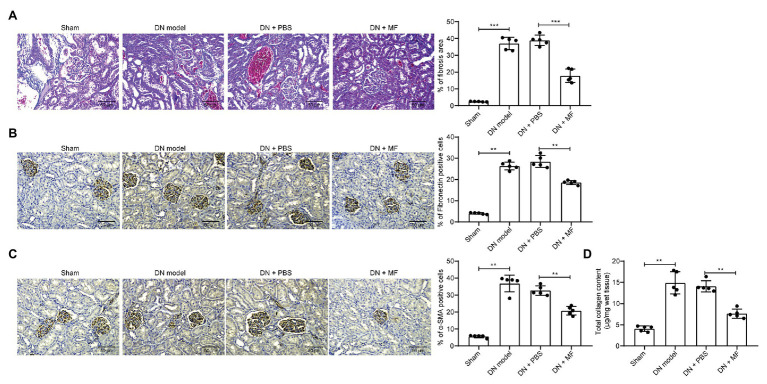
MF attenuates STZ-induced fibrosis and extracellular matrix (ECM) accumulation in rat renal tissues. **(A)** fibrosis levels in renal tissue by Masson’s staining; **(B)** the levels of fibronectin in renal tissues examined by immunohistochemistry; **(C)** the levels of alpha skeletal muscle actin (α-SMA) in renal tissues examined by immunohistochemistry; and **(D)** total collagen content detected by Collagen kits. All experiments were repeated three times. Values are expressed as mean ± SD (*n* = 5, each dot represents a rat in **A–D**). Variance among more than two groups was analyzed by one-way ANOVA, followed by Tukey’s *post hoc* test. ^**^p < 0.01, ^***^p < 0.001.

Moreover, we observed by Masson’s staining that STZ induced fibrosis (Figure 2A) in rat kidney. It was also revealed by immunohistochemistry that the expression of fibrosis-related factors fibronectin and *α*-SMA in tissues was enhanced (Figures 2B,C). After we treated rats with 100 mg/kg MF, the level of fibrosis in the renal tissues of the rats was reduced (Figures 2A–C), indicating that MF has the potency to alleviate STZ-induced DN in rats. Moreover, the collagen kit was used to detect the total collagen content in the tissue, and the accumulation of extracellular matrix (ECM) was significantly inhibited after treatment with MF (Figure 2D).

### MF Reduces the Proliferation of Mesangial Cells Caused by HG

**Figure 3:**
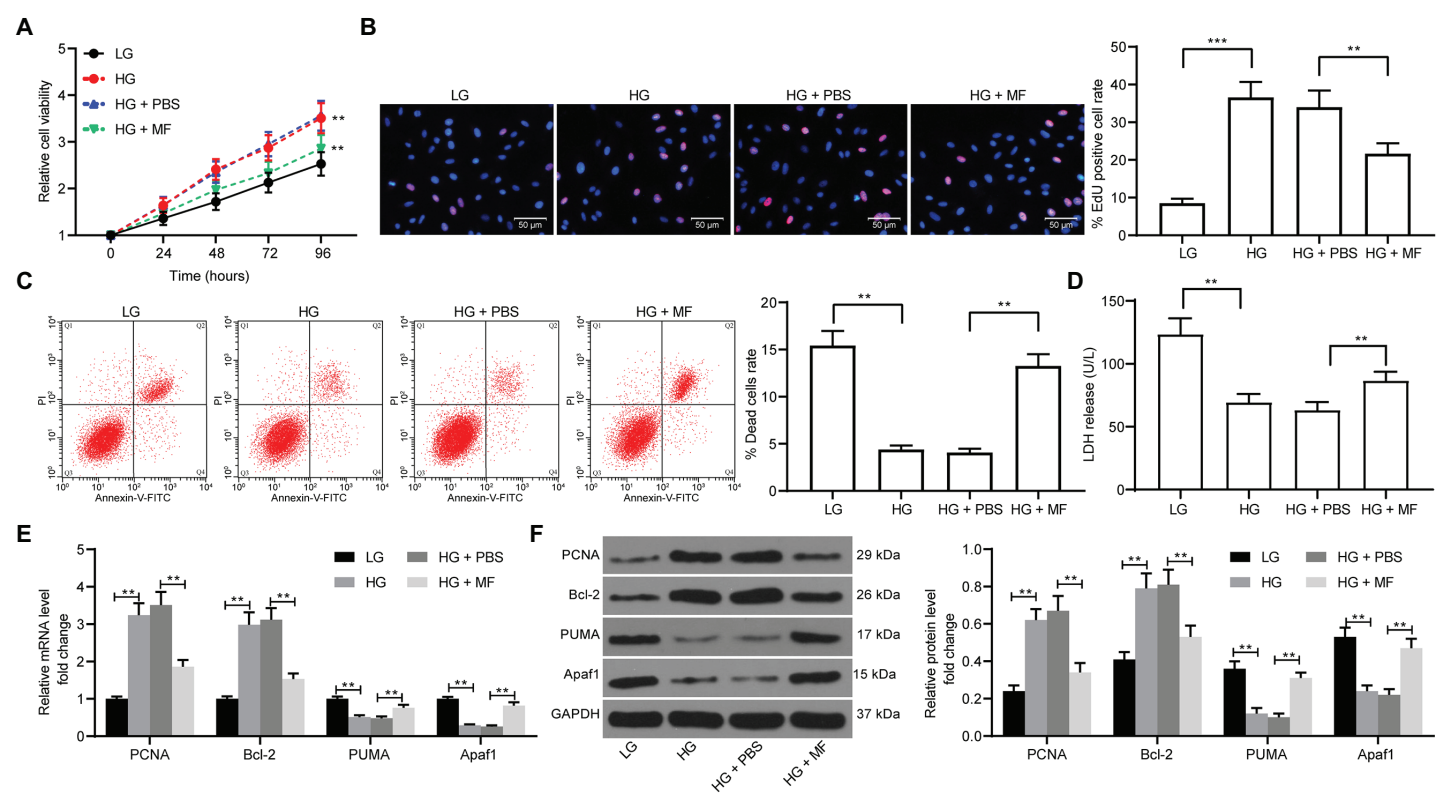
MF treatment reduces the proliferation of mesangial cells caused by high glucose (HG) treatment. Mesangial cells were exposed under HG to induce a diabetic renal injury cell model *in vitro* and treated with 100 mg/ml MF. **(A)** detection of cell proliferation by cell counting kit-8 (CCK-8) assays; **(B)** ethynyl-2'-deoxyuridine (EdU)-positive cells in mesangial cells; **(C)** flow cytometric analysis of mesangial cell death; **(D)** lactate dehydrogenase (LDH) release levels in mesangial cells determined by kits; **(E)** reverse transcription-quantitative polymerase chain reaction (RT-qPCR) detection of the messenger RNA (mRNA) expression of apoptosis-related factors in cells; and **(F)** western blot detection of the protein expression of apoptosis-related factors in cells. All experiments were repeated three times. Values are expressed as mean ± SD. Variance among more than two groups was analyzed by one-way (**B–D**) or two-way ANOVA (**A,E,F**), followed by Tukey’s *post hoc* test. ^**^p < 0.01, ^***^p < 0.001.

To further validate the role of MF in injury *in vitro*, we constructed a cell model by using HG-induced mesangial cells and treated cells with 100 mg/ml MF. We first used CCK-8 assays to detect the proliferation of cells. The proliferative ability of mesangial cells was obviously promoted after HG induction, but after further treatment with MF, the proliferation rate was significantly inhibited (Figure 3A). Moreover, we further used EdU staining to detect cell proliferation. The results were consistent with CCK-8 where MF treatment significantly reduced the number of red cells (Figure 3B). Furthermore, the proportion of apoptosis was detected by flow cytometry. We found that HG treatment significantly inhibited the death of mesangial cells, but after further treatment with MF, the cell death was increased significantly (Figure 3C). Moreover, we used LDH kits to detect the LDH activity released by cells. The release of LDH was significantly reduced after HG treatment, while MF treatment increased LDH release (Figure 3D). We then used RT-qPCR and western blot to assess the expression of apoptosis-related factors (PCNA, PUMA, Apaf1, and Bcl-2) in cells. The expression of PCNA and Bcl-2 in cells was increased after HG treatment, whereas the expression of PUMA and Apaf1 was significantly inhibited. Further, application of MF reversed these trends (Figures 3E,F).

### MF Treatment Attenuates Inflammation and Fibrosis in Mesangial Cells

**Figure 4:**
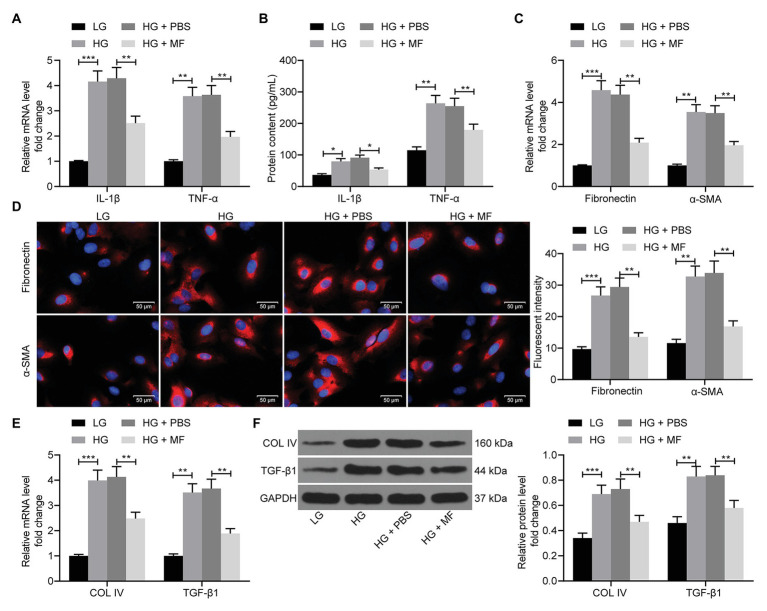
MF attenuates inflammation and fibrosis in mesangial cells caused by HG. **(A)** RT-qPCR detection of the mRNA expression of IL-1β and TNF-α in cells; **(B)** western blot detection of the protein expression of IL-1β and TNF-α in cells; **(C)** RT-qPCR detection of the mRNA expression of fibronectin and α-SMA in cells; **(D)** the immunofluorescence staining of fibronectin and α-SMA in cells; **(E)** RT-qPCR detection of the mRNA expression of Collagen IV and TGF-β1 in cells; and **(F)** western blot detection of the protein expression of collagen IV and TGF-β1 in cells. All experiments were repeated three times. Values are expressed as mean ± SD. Variance among more than two groups was compared by two-way ANOVA, followed by Tukey’s *post hoc* test. ^**^p < 0.01, ^***^p < 0.001.

Subsequently, RT-qPCR, ELISA, and western blot were performed to evaluate the expression of inflammation-, fibrosis-, and ECM-related factors in cells. The levels of IL-1β, TNF-α (Figures 4A,B), fibronectin, α-SMA (Figures 4C,D), collagen IV, and TGF-β1 (Figures 4E,F) in mesangial cells were increased significantly after HG treatment. However, following MF treatment, the levels of pro-inflammatory factors in mesangial cells repressed significantly, accompanied by lowered expression of fibronectin and α-SMA, and decreased aggregation of ECM (Figures 4A–F).

### MF Promotes Ubiquitination and Degradation of KDM3A

**Figure 5:**
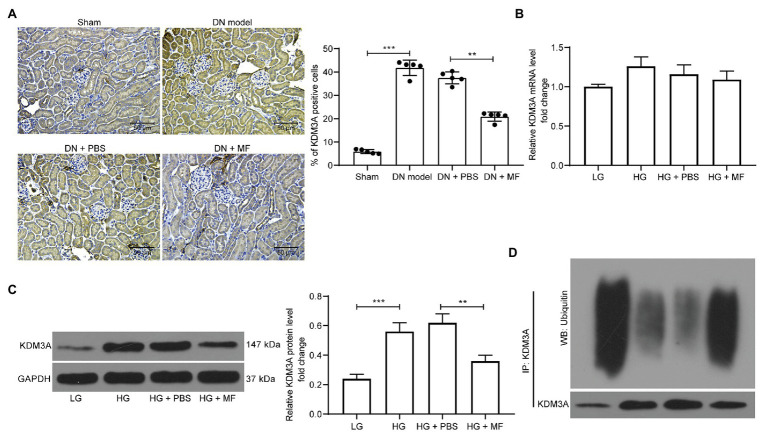
MF promotes ubiquitination and degradation of lysine-specific demethylase 3A (KDM3A). **(A)** immunohistochemical staining of KDM3A in renal tissues of rats in each group; **(B)** RT-qPCR detection of the mRNA expression of KDM3A in cells; **(C)** western blot detection of the protein expression of KDM3A in cells; and **(D)** detection of KDM3A ubiquitination in mesangial cells by Co-Immunoprecipitation (Co-IP). All experiments were repeated three times. Values are expressed as mean ± SD. Variance among more than two groups was analyzed by one-way ANOVA, followed by Tukey’s *post hoc* test. ^**^p < 0.01, ^***^p < 0.001.

It has been previously shown that Honokoil promoted the ubiquitination and degradation of oncogenic protein AML1-ETO, thus promoting apoptosis in leukemia cells (Zhou et al., 2017). Moreover, silencing of KDM3A inhibited renal damage caused by HG treatment (Shao et al., 2020). Hence, we speculated that MF might alleviate DN by promoting KDM3A ubiquitination and degradation. Therefore, we assessed the expression of KDM3A in rat renal tissues by immunohistochemistry, and noted that STZ treatment significantly promoted the expression of KDM3A in renal tissues. After MF treatment, the number of KDM3A-positive cells was significantly reduced (Figure 5A). Subsequently, we detected KDM3A mRNA and protein expression in mesangial cells. HG was observed to result in a significant increase in KDM3A mRNA and protein expression in mesangial cells. However, after MF treatment, KDM3A protein levels decreased significantly without significant changes in mRNA levels (Figures 5B,C). Thus, we used Co-Immunoprecipitation (Co-IP) to detect the KDM3A protein ubiquitination in mesangial cells. The level of KDM3A ubiquitination was significantly increased (Figure 5D) after MF treatment.

### MG132 Treatment Weakens the Protective Effect of MF on Mesangial Cells

**Figure 6:**
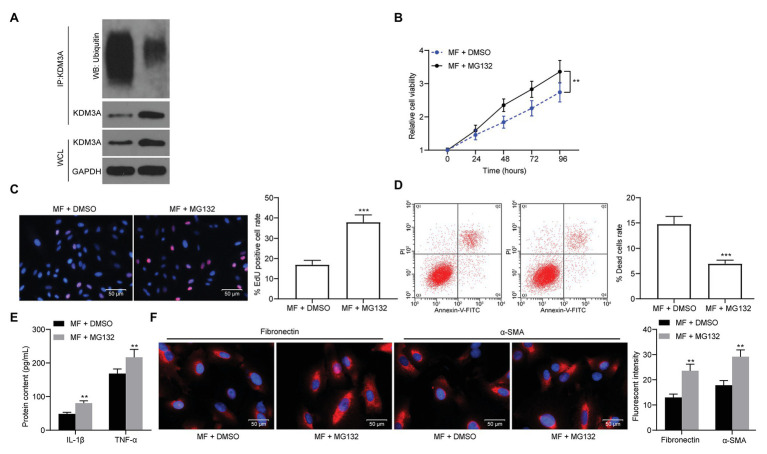
MG132 treatment weakens the protective effect of MF on mesangial cells. A proteasome inhibitor, MG132, was added to MF-treated cells. **(A)** detection of KDM3A ubiquitination in mesangial cells after MG132 treatment; **(B)** detection of cell proliferation by CCK-8 assays; **(C)** EdU-positive cells in mesangial cells; **(D)** flow cytometric analysis of mesangial cell death; **(E)** ELISA detection of the expression of IL-1β and TNF-α in cells; and **(F)** the immunofluorescence staining of fibronectin and α-SMA in cells. Data between two groups were analyzed using the unpaired *t*-test (**C**,**D**), whereas variance among more than two groups was analyzed by two-way ANOVA (**B**,**E**,**F**), followed by Tukey’s *post hoc* test. ^**^p < 0.01, ^***^p < 0.001.

To validate our conjecture, we added a proteasome inhibitor MG132 to MF-treated cells. After the addition of MG132, KDM3A ubiquitination levels were significantly reduced (Figure 6A). Meanwhile, the activity of mesangial cells was significantly promoted, and cell apoptosis or death caused by MF treatment was significantly inhibited after the addition of MG132 treatment (Figures 6B–D). Moreover, MG132 contributed to promoted levels of pro-inflammatory and pro-fibrotic proteins (Figures 6E,F).

### KDM3A Inhibits TGIF1 Activity and Promotes TGF-β1/Smad2/3 Signaling Activation

**Figure 7:**
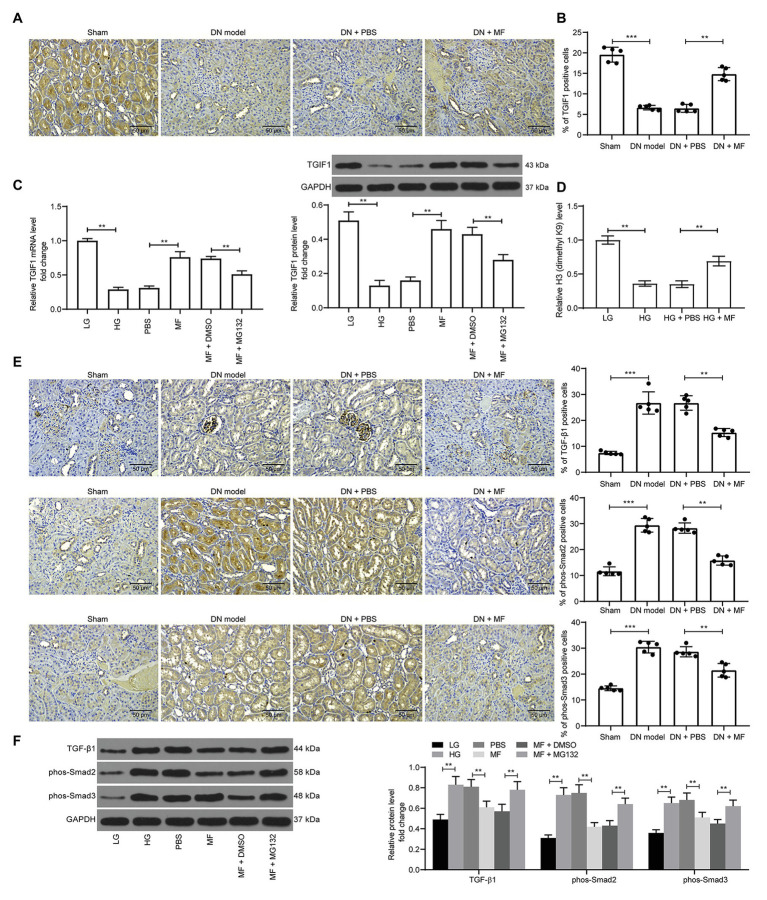
KDM3A inhibits transforming growth factor β-induced factor 1 (TGIF1) activity and promotes the TGF-β1/Smad2/3 signaling activation. **(A)** immunohistochemical staining of TGIF1 in renal tissues of rats in each group; **(B)** RT-qPCR detection of the mRNA expression of TGIF1 in cells; **(C)** western blot detection of the protein expression of TGIF1 in cells; **(D)** chromatin immunoprecipitation (ChIP)-qPCR detection of the level of H3K9me2 modification of the TGIF1 promoter in mesangial cells; **(E)** immunohistochemical staining of TGF-β1 expression and Smad2/3 phosphorylation in renal tissues of rats in each group; **(F)** western blot detection of TGF-β1 expression and Smad2/3 phosphorylation levels. Variance among more than two groups was analyzed by one-way (**A–C**) or two-way ANOVA (**D–F**), followed by Tukey’s *post hoc* test. ^**^p < 0.01, ^***^p < 0.001.

In a previous study, microRNA-101 inhibited renal fibrosis by inhibiting KDM3A expression to regulate TGIF1 expression (Ding et al., 2020). Thus, we first examined the TGIF1 expression in rat kidney and found that STZ treatment significantly inhibited the expression of TGIF1 in the kidney (Figure 7A). Similarly, in mesangial cells treated with HG, the expression of TGIF1 was significantly inhibited, while the expression of TGIF1 was remarkably increased after the addition of MF. After further addition of MG132, the expression of TGIF1 was significantly decreased (Figures 7B,C). We further used ChIP-qPCR to detect the level of H3K9me2 modification of the promoter sequence of TGIF1 in mesangial cells, and we found a significant decline in H3K9me2 levels of TGIF1 in the cells after HG exposure, but a significant increase in H3K9me2 levels after further treatment with MF (Figure 7D). More specifically, TGIF blocked the activation of the TGF-β1/Smad2/3 signaling pathway (Guca et al., 2018). We then used immunohistochemistry to detect the expression of TGF-β1 and Smad2/3 in rat renal tissues. After STZ induction, the expression of TGF-β1 in rat renal tissues was increased, and the phosphorylation level of Smad2/3 was elevated. After the addition of MF, the TGF-β1 expression and Smad2/3 phosphorylation were significantly decreased (Figure 7E). Consistent results were also observed in mesangial cells. In addition, MG132 administration comparably induced the TGF-β1 expression and Smad2/3 phosphorylation (Figure 7F).

### Knockdown of TGIF1 Reverses the Decline in Activity of Mesangial Cells Caused by MF

**Figure 8:**
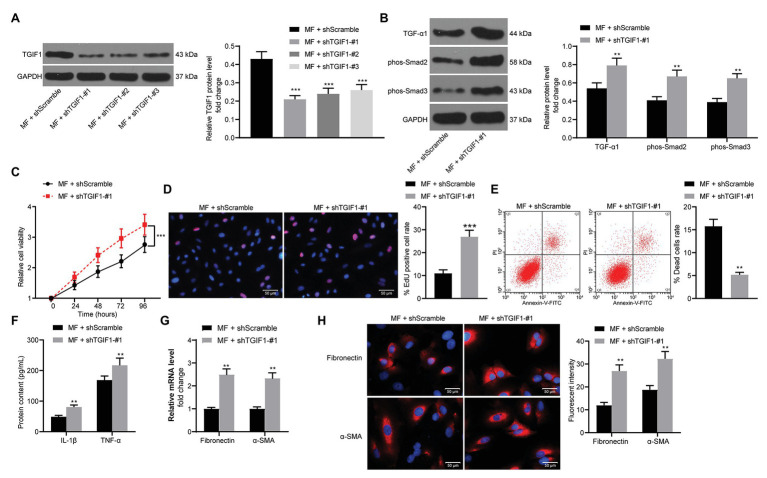
Knockdown of TGIF1 promotes the mesangial cell activity. **(A)** western blot detection of TGIF1 protein expression in cells; **(B)** western blot detection of TGF-β1 expression and Smad2/3 phosphorylation levels; **(C)** detection of cell proliferation by CCK-8 assays; **(D)** EdU-positive cells in mesangial cells; **(E)** flow cytometric analysis of mesangial cell death; **(F)** ELISA detection of the expression of IL-1β and TNF-α in cells; **(G)** RT-qPCR detection of fibronectin and α-SMA in cells; and **(H)** the immunofluorescence staining of fibronectin and α-SMA in cells. Data between two groups were compared using the unpaired *t*-test (**A**,**D–H**), whereas variance among more than two groups was analyzed by two-way ANOVA (**B**,**C**), followed by Tukey’s *post hoc* test. ^**^p < 0.01, ^***^p < 0.001.

Subsequently, we transfected three shRNAs targeting TGIF1 into MF-treated mesangial cells. Western blot verified the successful development of mesangial cells with low expression TGIF1 (Figure 8A). We first examined TGF-β1 expression and Smad2/3 phosphorylation in cells. It was found that knocking-down TGIF1 expression in mesangial cells significantly promoted TGF-β1 expression and Smad2/3 phosphorylation (Figure 8B). Furthermore, after TGIF1 knockdown, the viability and proliferation of mesangial cells were remarkably increased (Figures 8C,D), and the proportion of cell death was significantly reduced (Figure 8E). After knocking-down TGIF1, the secretion of inflammatory factors in the cells augmented (Figure 8F), and the expression of fibrosis-related factors increased significantly (Figures 8G,H). It was thus suggested that the attenuation of inflammatory responses and fibrosis was achieved *via* TGIF1 activity enhancement and inhibition of TGF-β1/Smad2/Smad3 pathway.

## Discussion

Diabetic nephropathy, occurring in 20–50% of all diabetic patients, is characterized by persistent albuminuria and a decline in renal function (Selby and Taal, 2020). Hyperglycemia and hyperfiltration contribute to morphological changes, such as mesangial expansion, ECM accumulation, and glomerulosclerosis with nodular mesangial lesion (Chang and Chen, 2020). Moreover, fibrosis has the characteristics of excessive deposits of ECM that leads to the replacement of functional parenchyma by fibrotic tissue, and renal fibrosis is the commonest pathological process in chronic kidney disease (Calle and Hotter, 2020). Therefore, the current study is conducted to uncover the mechanisms of MF in DN with the conclusion pronouncing that MF inhibits inflammatory response and fibrosis in DN progression *via* the KDM3A/TGIF1/TGF-*β*1/Smad2/3 axis.

In this study, SD rats were subjected to an intraperitoneal injection of STZ to induce DN. Renal tissue section staining showed obvious hyperplasia, increased infiltration of inflammatory cells, and blurred space between the cells, which was consistent with characteristics of early DN. This indicated the successful development of a rat model with DN. MF, a secondary metabolite that is generated in the metabolic process of plants, exerts both antidiabetic and anti-inflammatory functions (Xu et al., 2020). Recently, it has been suggested to induce autophagy and apoptosis of gastric and breast cancer cells, thereby participating in the progression of cancers (Sun et al., 2020a; Wei et al., 2020). On top of that, MF has been indicated to inhibit apoptosis of keratinocytes to treat *Atopic dermatitis*, a chronic skin disease inflammatory (Wu et al., 2020). MF could also effectively repress inflammatory calvarial osteolysis in mice and suppress inflammation as well as osteoclastogenesis *in vitro* (Sun et al., 2020b), indicating that the possible association between MF and inflammatory response. Consistently, our *in vitro* and *in vivo* evidence both proposed that MF lowered the levels of IL-1β and TNF-α in serum of rats with renal injury induced by STZ and mesangial cells exposed to HG. Nevertheless, the linkage between MF and fibrosis has rarely investigated. We established here that MF reduced fibronectin and α-SMA expression *in vivo* and *in vitro*.

Furthermore, myocardin-related transcription factor A was found to interact with and recruit histone demethylase KDM3A to the promoter of connective tissue growth factor to activate its transcription, and KDM3A silencing weakened HG-induced connective tissue growth factor activation in renal tubular epithelial cells (Shao et al., 2020). Similarly, we observed that MF promoted ubiquitination and degradation of KDM3A. To verify our postulation, a recue experiment was carried out in MF-treated cells using a proteasome inhibitor MG132 which significantly promoted cell viability and the levels of pro-inflammatory and fibrotic factors. In line with our study, MG132 could provide renoprotection by repressing Akt-dependent inflammation in DN (Zeng et al., 2019). TGIF1 has the potency to inhibit TGF-induced transcription through interacting with Smad2-Smad4 complexes (Guca et al., 2018). Otitis media are accompanied by raised expression of vascular endothelial growth factor, TNF-α and IL-1β in ear fluids, during which TGIF1 mutants illustrate auditory deficits (Tateossian et al., 2013). Also, in lung transplant biopsies from patients with bronchiolitis obliterans syndrome, an increase in miR-144 was noted to downregulate TGIF1, thereby elevating TGFβ secretion to contribute to increased fibrosis (Xu et al., 2015).

The increased expression of TGF-β1 and p-Smad3/Smad3 was identified in mesangial cells exposed to HG (Li et al., 2017; Fan et al., 2020), which was largely in line with our *in vitro* data. Moreover, we observed consistent results in rats challenged with STZ, corroborating the activation of the TGF-β1/Smad2/Smad3 signaling in DN development. The paramount importance of the TGF-β1/Smad signaling has been underscored in various DN pathogenesis, includes podocyte injury, mesangial cell proliferation, and especially renal fibrosis (Isacsson et al., 1988; Tang et al., 2018a,b, 2020; Zhang et al., 2019; Qi et al., 2020). As an important regulator in the pathway, TGF-β1 has been recognized as an effective cytokine that modulates cell proliferation, differentiation, migration, and ECM turnover in the kidney (Lan and Chung, 2012). However, the association between TGF-β1/Smad2/Smad3 and MF has never been investigated. Here, we found that MF impaired the activation of the TGF-β1/Smad2/Smad3 pathway, which was restored by MG132, which suggested that MF-mediated KDM3A was indeed an upstream biomolecule of the pathway.

## Conclusion

In conclusion, the findings illustrate that MF possesses properties that attenuate DN and suppress inflammatory responses and fibrosis. In addition, the repressive effects were accomplished by the ubiquitination and degradation of KDM3A and the downstream augment of TGIF1 and the TGF-β1/Smad2/Smad3 signaling deficit. Altogether, these results indicate that MF might be potentially applied to prevent or treat DN as well as other diabetic diseases associated with inflammation. However, further studies involving MF as an antiglycemic drug ought to be warrant for clinical translation in future.

## Data Availability Statement

The original contributions presented in the study are included in the article/supplementary material, further inquiries can be directed to the corresponding author.

## Ethics Statement

The animal study was reviewed and approved by the Ethics Committee of the First Affiliated Hospital of Harbin Medical University.

## Author Contributions

LC and QW are the guarantors of integrity of the entire study and contributed to the concepts and design of this study. JJ and YL contributed to the experimental studies. QC contributed to the data and statistical analysis. LH took charge of the manuscript preparation. YZ contributed to the manuscript review. All authors contributed to the article and approved the submitted version.

### Conflict of Interest

The authors declare that the research was conducted in the absence of any commercial or financial relationships that could be construed as a potential conflict of interest.
